# Application of Proteomic Technologies to Assess the Quality of Raw Pork and Pork Products: An Overview from Farm-To-Fork

**DOI:** 10.3390/biology9110393

**Published:** 2020-11-11

**Authors:** María López-Pedrouso, José M. Lorenzo, Mohammed Gagaoua, Daniel Franco

**Affiliations:** 1 Department of Zoology, Genetics and Physical Anthropology, University of Santiago de Compostela, 15872 Santiago de Compostela, Spain; mariadolores.lopez@usc.es; 2 Centro Tecnológico de la Carne de Galicia, Rúa Galicia N° 4, Parque Tecnológico de Galicia, 32900 San Cibrao das Viñas, Spain; jmlorenzo@ceteca.net; 3 Área de Tecnología de los Alimentos, Facultad de Ciencias de Ourense, Universidad de Vigo, 32004 Ourense, Spain; 4 Food Quality and Sensory Science Department, Teagasc Ashtown Food Research Centre, Ashtown, D15 DY05 Dublin 15, Ireland; gmber2001@yahoo.fr

**Keywords:** pig, high-throughput proteomic tools, meat quality, molecular biomarkers, traceability, authentication

## Abstract

**Simple Summary:**

The swine industry has an important interest in becoming more efficient and innovative, fitting with the consumer demand in terms of pork quality, meat pig welfare conditions, and the environmental aspects. This paper deals with the productive (breed, diet, stress) and technological (aging, cooking) factors that affect fresh pork and elaborates the quality of products by using proteomic tools for assessing. These technologies are a relevant approach in the meat science field to decipher the underlying mechanisms and post-mortem changes in the muscle and biofluids proteome of pigs because their study will allow better management of the outcomes such as meat quality variation and defects. In general, these new developments in molecular techniques can help researchers to control and assess this quality through biomarkers. Additionally, as food safety and pork product authentication/adulteration to avoid fraud can be evaluated with these high-throughput proteomic tools, hence they were tackled. Finally, another relevant point addressed focused on the search of bioactive peptides with a beneficial effect on human health from added-value products such as dry-cured ham. Overall, this review describes the current and emerging proteomics studies dealing with raw pork and pork products from the farm to fork.

**Abstract:**

The quality assurance of pork meat and products includes the study of factors prior to slaughter such as handling practices, diet and castration, and others during the post-mortem period such as aging, storage, and cooking. The development over the last two decades of high-throughput techniques such as proteomics offer great opportunities to examine the molecular mechanisms and study a priori the proteins in the living pigs and main post-mortem changes and post-translational modifications during the conversion of the muscle into the meat. When the most traditional crossbreeding and rearing strategies to improve pork quality were assessed, the main findings indicate that metabolic pathways early post-mortem were affected. Among the factors, it is well documented that pre-slaughter stress provokes substantial changes in the pork proteome that led to defective meat, and consequently, novel protein biomarkers should be identified and validated. Additionally, modifications in pork proteins had a strong effect on the sensory attributes due to the impact of processing, either physical or chemical. Maillard compounds and protein oxidation should be monitored in order to control proteolysis and volatile compounds. Beyond this, the search of bioactive peptides is becoming a paramount goal of the food and nutraceutical industry. In this regard, peptidomics is a major tool to identify and quantify these peptides with beneficial effects for human health.

## 1. Introduction

The global demand and industrial production of pork and pork products are increasing. According to the Food and Agriculture Organization of the United Nations (FAO) statistics, worldwide swine production has increased steadily from 1961 to 2007, with Asia the continent with the highest production with around 50% of the total worldwide production. Between 2007 and 2017, production increased significantly in Asia and remained as the highest swine producers (almost 60% of the world total), since China assumed most of the production with more than 3700 million pigs produced during this time. Europe, with more than 25% of world production, is the second continent with the highest production. Germany, Poland, and Spain are the major countries with a remarkable rate in swine production [1]. Regarding pork consumption, the Asian continent is the area of the world with the greatest increase, from 2.8 million tons in 1961 to 67.4 in 2013, mainly related to China. For example, in 2013, pork demand in this country accounted for 80% of the Asian continent and around 50% worldwide [1].

From the above, it seems that the swine industry has significant interest in becoming more efficient and innovatively fitting with the consumer demand. Consumers are now demanding higher standards for the welfare of pigs and pork quality. Factors affecting the production system (breed, age/slaughter weight, sex, castration, and diet), pre-slaughter conditions (fasting, transport, lairage and stunning), and post-mortem interventions (electrical stimulation of the carcasses, aging, and storage conditions in terms of time/temperature, cooking, etc.) have an impact on pork eating quality, therefore, these factors should be carefully monitored during the whole continuum of farm-to-fork, especially at the industrial level. The relationship between animal welfare and pork quality has been detailed and rigorously researched from a proteomic point of view [2]. During the processing of meat, tenderness, color, and water holding capacity (WHC) traits are strongly impacted. Indeed, tenderness, intramuscular fat, cooking loss, and sensory traits are the main qualities of pork meat, which have been the most investigated and improved during the last decades. Currently, the meat research is focusing on a deeper understanding of the conversion of muscle into meat. This process results from myriad interconnected pathways including the enzymatic action of endogenous proteolytic enzymes of the muscle such as calpains; afterward, the impact of pH decline and that of lysosomal enzymes (cathepsins) that break the supramolecular structure of sarcomere as well as weakening the Z-discs, hence reducing the strength of the anchoring of the actomyosin complex and myofibrillar proteins [3]. In these stages, protein degradation plays an important role in the development of the pork quality traits (pH, color, WHC, and tenderness), causing when uncontrolled, serious technological defects and economic losses to occur [4].

One of the main problems associated with poor pork quality is the PSE (pale, soft, exudative) meat defect. This defect can be, for example, exacerbated by inadequate mixing with animals from different pens or batches raising fights and aggressions, increasing their stress pre-slaughter, hence causing PSE meat [5]. Stressed pigs are associated with a higher concentration of lactate and rapid pH decline during rigor mortis, provoking PSE meat [6]. Moreover, long journeys could result in lower carcass pH at 45 min, leading to a high incidence of PSE meat [7]. Along the same line, an inadequate carcass cooling process could modify the metabolic processes and the extent of pH decline, hence leading to PSE meat [5]. In the case of PSE meat, changes in the sarcoplasmic and myofibrillar proteins are observed, and specifically, a lower solubility and higher denaturation of the proteins are evidenced [8]. This phenomenon could be the result of several biological pathways such as fast post-mortem glycolysis in the muscle, which are specifically targeted by activation of glycogen phosphorylase and phosphofructokinase, reducing the pH and protein denaturation and therefore inducing the PSE defect [9,10]. Consequently, pig stress has adverse consequences on fresh pork due to the impact on the transformation of muscle into meat. Considering the above, this is a major problem to the industry due to the economic losses, as this type of meat cannot be used for the elaboration of high-value cured meat products and other products. In this sense, a prediction of PSE through protein modifications would be a powerful strategy [11], among other emerging chemometric methods [10].

**Figure 1:**
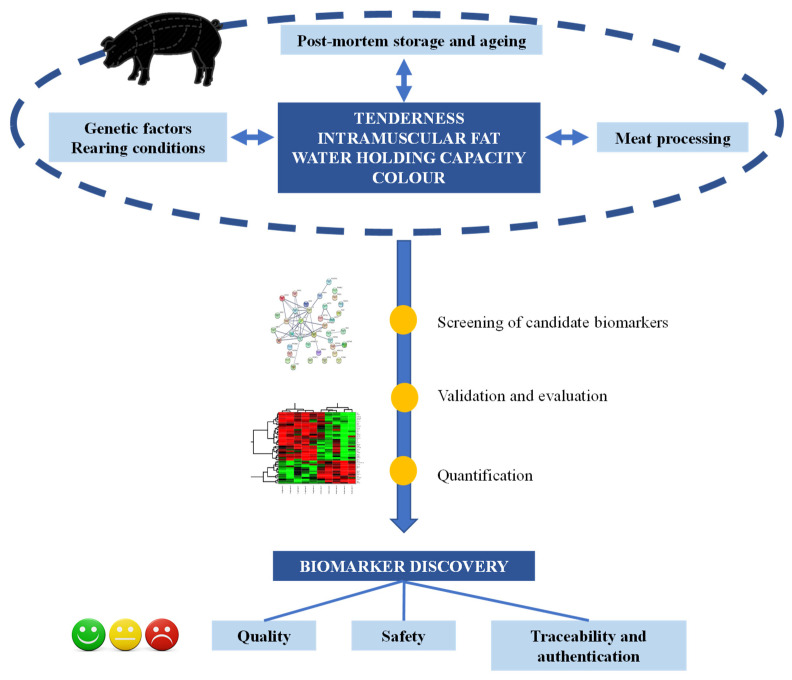
Proteomic workflow in the search for protein biomarkers regarding pork quality.

In the framework of high-throughput omics technologies, proteomics offers insights about the complex network of proteins and pathways underlying variation in pork quality. The study of the post-mortem muscle proteome together with protein–protein interactions, and post-translational modifications of proteins become a challenging task to deliver high-quality meat products [12]. In particular, the proteome is the result of a gene expression influenced by environmental and processing conditions related to the functional quality characteristics of the meat [13]. Gel-based proteomics and analytical approaches based on mass spectrometry are increasingly being used. In the proteomic workflow, often the initial step is a fraction of protein extracts separated using gel electrophoresis [one dimensional Sodium Dodecyl Sulfate Polyacrylamide Gel Electrophoresis (SDS-PAGE) or two-dimensional gel electrophoresis (2-DE)] coupled to liquid chromatography tools for identification. The large capacity and power of these technologies to study plant and animal products has been demonstrated [14,15,16,17]. Afterward, the bottom-up approach is the most common strategy resulting from the extraction of proteins and digestion by sequence-specific enzymes for later analysis by mass spectrometry [18]. It should be highlighted that those post-translational modifications of the proteins and their interactions with other proteins or macromolecules have a strong impact on pork tissues because of the change in the three-dimensional structure and consequently of the cellular functions [19]. Indeed, solubility, thermal stability, gelation, emulsifying, foaming, fat binging, and water-binding are only a few of parameters that directly depend on the protein structure, resulting in its great importance in food science [20]. For all these reasons, food proteomics, also known as foodomics [21], provide a great opportunity in the quality and safety controls of pork and pork products (Figure 1).

Therefore, this review aimed to provide a summary of the current pork proteomics studies with the aim for a better understanding of the protein changes and their relationships with quality traits and the processing parameters of pork and its associated products. Thus, current technological advances and their integration with other traditional techniques are further briefly discussed. This review includes a current collection and non-exhaustive list of papers in the field, which have been discussed.

## 2. Proteomic Perspective of Breeding and Rearing Practices

For consumers, the perception of muscle color, texture, and marbling are the main aspects that drive their choice. The consumer’s first impression is strongly associated with the pork color at the point of sale whereas muscle firmness/texture and marbling are used to predict the final eating quality. This complex network of sensory attributes defined by color, pH, texture, water holding capacity, and marbling can be, for example, impacted by breed selection and rearing practices [22]. In general, meat quality traits are the result of genetic and environmental interactions. Therefore, livestock production systems including feeding, housing conditions, genotypes, behavioral, and physiological responses are widely recognized as the key factors driving pork quality. In recent years, different strategies aiming to improve the pork quality such as breed selection, animal management, and feeding, and other aspects of pork processing have been considered. Some recent studies in this context are briefly summarized in Table 1. Proteomics has mainly been employed to evaluate the meat quality by searching protein biomarkers and defining the molecular pathways as reviewed by Schilling et al. [4]. The great majority of proteomic experimental designs have been aimed to understand the underlying biological processes and identify biomarkers for meat processing [23]. 

### 2.1. Influence of Pig Breed-Specific Differences among Genotypes for Final Pork Quality

From a proteomic point of view, pig breed-specific differences among genotypes have shown different growth performances and fat accumulation as well as variations in the anaerobic activity of pig muscles after slaughter. These facts allow for explaining how differences in post-mortem temperature among breeds provoke differences in the activity of calpains and cathepsins, resulting in tender or tough meat [24]. Novel and traditional breeds are being developed to increase both productivity and pork quality. For instance, proteome analysis has proven that proteins associated with meat quality such as calcium ion binding proteins, Calsarcin 1, carbonic anhydrase 1 (CA1), myosin-binding protein H (MYBPH), and other metabolic proteins were differentially expressed between Tibetan (local) and Duroc × (Landrace × Yorkshire) (commercial) breeds. Meanwhile, the enrichment of Gene Ontology (GO) showed the purine-containing compound metabolic process as the molecular function, threonine-type peptidase activity as the molecular function, and mitochondrial inner membrane as the cellular component were the key aspects in the differentiation among these breeds [25]. Similarly, in another study that aimed to search for differences in the proteome of the Shaziling (local) versus Yorkshire breed, the GO term enrichment displayed the importance of metabolism, protein binding, and the regulation of skeletal muscle development within the differential proteins [26]. In both studies underlying the breed effect and differences in the proteome, the metabolic proteins were affected by genotype, indicating that the muscle fiber types were altered, and therefore, their metabolic properties. It has been demonstrated that metabolic properties influence the conversion of muscle to meat and meat quality in the early stages [27,28], but post-mortem metabolic processes have a higher influence on tenderness, water holding capacity, and technological traits [29].

### 2.2. Influence of Feed Efficiency on Pig Production

Another relevant aspect of pig production is the residual feed intake (RFI) used to calculate the feed efficiency of growing animals at the farm. This trait may also be predicted using a proteomic approach as RFI can be correlated with molecular differences in skeletal muscle such as composition and muscle characteristics or other protein fluids such as plasma [30,31]. In this sense, low RFI is, for instance, associated with a greater proportion of fast-twitch glycolytic fibers characterized by higher glycolytic activity in comparison with high RFI [32]. Regarding metabolic pathways in skeletal muscle, proteomic studies have revealed that mitochondrial energy is lower for high feed efficiency. Moreover, the glucose–pyruvate–TCA–oxidative phosphorylation pathway has been identified as one of the key pathways of feed efficiency in pigs [31]. Vincent et al. [33] found similar results as the mitochondrial oxidative proteins had a lower abundance in pig muscles with lower RFI diet, reflecting a lower muscular oxidative stress. On the contrary, proteins involved in the glycolysis pathway had a higher abundance in pigs from a low-RFI (“efficient”) compared with those from high-RFI pigs (“inefficient”). Another way to increase the feed and growth rate, carcass yield, and carcass leanness is to feed pigs with additives such as ractopamine. Under these conditions, the abundance of enzymes involved in glycolytic metabolism are also modified, producing alterations in the conversion of muscle to meat [34]. Even improving intramuscular fat by changing the pig diet provokes differential expression in the proteins belonging to pathways like energy metabolism, fiber type, and structure, specifically slow-twitch troponin 1 and myosin heavy chain IIb [35]. It may be inferred that different dietary conditions may lead to variations in muscle fiber type composition, and consequently, the different glycogen and lactate contents. As explained previously, changes in glycogen and lactate concentrations at the early post-mortem stage may impact meat quality and if excessive changes lead to defects in the final meat products [36].

### 2.3. Influence of the Castration on Pork Quality

On the other hand, within the swine industry, a widespread practice employed is the castration of male piglets. The main reasons why the pigs are castrated are an improvement in pig management, growth, carcass qualities, and mainly to avoid the unpleasant aroma and taste of pork perception related to “boar taint”. This latter reason is probably the most important due to the odor and flavor characteristics in raw and cooked pork meat, which causes rejection. However, the controversy about the surgical castration of pigs has led to the search for alternatives such as immunocastration. This option consists of vaccinating against gonadotropin-releasing factor (GnRH). However, there is little research about the effect of immunocastration on meat quality [37]. Using the LC-MS/MS approach, the proteomic differences among intact males and surgically (SC) and immunologically castrated (IC) pigs were revealed [38]. A total of 50 proteins were differentially abundant between the IC and SC treatments. Twenty-two proteins were greater in abundance for the IC group, while 27 with an abundance change fold higher than 1.5 varied between both castration methods studied. These findings suggest at first glance a strong relationship with pork quality development. In fact, the entire males showed an increase in oxidative metabolic profile than in the SC group. Furthermore, proteins involved in the cytoskeleton and immunity were abundant in the IC group, while several heat shock proteins and laminins were abundant in the SC group. In addition, the main structural proteins (actin and myosin heavy chain isoforms) were importantly degraded in the case of the entire animals in comparison to castrated pigs, although this was not a driver of more tender meat [39]. In a recent integromics study by Gagaoua and co-workers on cattle, the results on pork were consistent with the beef tenderness and color [14,17]. Briefly, the oxidative damage occurring in post-mortem muscle impacts the state of the structural proteins and increases the role of antioxidant and heat shock proteins that positively or negatively drive the final outcomes of these meat quality traits. Furthermore, in terms of the mechanisms, it seems that changes in the proteins of structure are related to other pathways such as oxidation, hence leading to the formation of protein aggregates that can negatively impact tenderness or color traits.

### 2.4. Relationship between Pre-Slaughter Stress and Pork Quality

The relationship between pre-slaughter stress and meat quality have been demonstrated, but several unknowns and more knowledge are needed at the molecular level, as reviewed by Mouzo et al. [2]. Apart from ethical considerations from consumers, pig stress largely creates PSE meat, causing economic losses, as indicated in the introduction. Pigs are particularly susceptible to heat stress because they lack sweat glands and skin insulation of subcutaneous fat. This major stressor provokes a metabolic and physiologic response. Additionally, certain pre-slaughter conditions such as mixing animals or long-term transportation increase the risk of the production of meat with high ultimate pH at the group level [40]. Studies looking at the relationship in detail showed that in pigs, levels of fighting were linearly related to increases in ultimate pH at the individual level [41]. At the molecular level, triosephosphate isomerase and transferrin have been proposed as biological markers for water holding capacity in the case of pigs under stress conditions [42]. In this regard, the hepatic proteome of pigs subjected to chronic heat stress was investigated, showing that proteins involved in response to stress (HSPs) and immune defense, response to oxidative stress, apoptosis, energy metabolism, signal transduction, and cytoskeleton were altered regardless of feeding. Overall, mechanisms of defense and homeostasis could be associated with heat stress, thereby contributing to the increase in meat shear force [43,44]. Specifically, the Halothane (HAL) genotype is characterized by suffering from a porcine stress syndrome, leading to the production of meat with poor quality such as PSE. The HAL mutation showed an upregulation of phosphorylation in proteins of calcium signaling, muscle contraction, glycogen, glucose and energy metabolism, and cellular stress, suggesting that among the proteins, Ca^2+^/calmodulin-dependent protein kinase II was a key enzyme with an interplay role in these processes [45]. Additionally, post-translational modifications of metabolic enzymes were detected by 2-DE. These modifications can be used as specific patterns in response to short- and long-term heat stress [46]. More specifically, protein acetylation has been investigated in relation to heat stress for further knowledge of the conversion of muscle in meat [47,48]. Protein acetylation, together with phosphorylation, has been recognized to regulate the activity of enzymes and the stability of proteins amongst other functions. It has been reported that preslaughter stress produces a dynamic protein lysine acetylation/deacetylation, which is associated with muscle contraction, carbohydrate metabolism, apoptosis, and calcium signaling [49]. Furthermore, individual housing also causes animal stress and this stressor was used as a means to search for stress biomarkers, as demonstrated by Marco-Ramel et al. [50]. For the above reasons, it can be concluded that pig stress is a major issue that has a great influence on muscle proteome, and thus of meat quality, as seen through the presence of different isoforms and important post-translational modifications during the post-mortem period.

**Table 1:** Recent studies regarding the effect of different production factors on improvements in pork quality.

Studied Factors		Protein Source	Proteomic Approach	Main Findings	Ref
Genotypes	Genotypes: Tibetan and Duroc × (Landrace × Yorkshire) (DLY). Feasibility of using differential proteomic analysis to discriminate among pig breeds and cuts	Different muscle cuts	LC-MS/MS	The differential proteins belong to two major categories: meat quality-associated proteins (calcium ion binding protein, Calsarcin 1, CA1 and MYBPH), and energy metabolism-associated proteins (CSRP3, GSTK1, COX6A, AMPD, and TXNL1). Regardless of pork cuts, comparative proteome analysis between Tibetan and DLY pork identified 102 differentially abundant proteins.	[25]
	Genotypes: Chinese indigenous Shaziling and the Yorkshire breeds	LD	2-DE and MALDI-TOF/TOF	23 differentially expressed proteins were identified and associated with fatty acid metabolism, glycolytic pathway, and skeletal muscle growth. These differentially expressed genes and proteins are candidate genes for improving meat quality in Shaziling pigs.	[26]
	Mechanisms of Halothane (HAL) and Rendement Napole (RN) Genes	LD	iTRAQ, TiO_2_ enrichment and LC-MS/MS	The HAL mutation contributes to the upregulation of phosphorylation in proteins related to calcium signaling, muscle contraction, glycogen, glucose and energy metabolism and cellular stress.	[45]
Feeding	Feed efficiency: high-FE and low-FE pigs were compared.	LD	iTRAQ LC-MS/MS	124 proteins were differentially expressed between the high- and low-FE pigs. The glucose–pyruvate–tricarboxylic acid–oxidative phosphorylation energy metabolism signaling pathway was an important regulated pathway. Enzymes involved in the conversion of glucose to pyruvate were upregulated in the high-FE pigs.	[31]
	Feed efficiency: Pigs with low-RFI (“efficient”) and high-RFI (“inefficient”)	LD	2-DE and LC-MS/MS	11 proteins showed a differential abundance between RFI lines. However, the differentially expressed proteins were not affected by feed restriction.	[33]
	Dietary ractopamine supplementation to improve pork leanness.	LD	2-DE and MALDI-TOF/TOF	Glyceraldehyde-3-phosphate dehydrogenase (GAPDH) and phosphoglucomutase-1 (PGM1) were over-abundant in control pigs, whereas serum albumin (ALB), carbonic anhydrase 3 (CA3), L-lactate dehydrogenase A chain (LDHA), fructose-bisphosphate aldolase A (ALDOA), and myosin light chain 1/3 (MYL1) were over-abundant in pigs ingested with ractopamine. Ractopamine suggested to influence the abundance of enzymes involved in glycolysis.	[34]
	Dietary L-arginine supplementation to reduce backfat thickness and increase intramuscular fat (IMF).	LD	2-DE and LC-MS/MS	The proteome changes in LD muscle between the control and supplemented pigs showed that L-arginine significantly influenced the abundance of proteins involved in energy metabolism, fiber type, and muscle structure.	[35]
Castration	Immune and surgical castrated pigs	LD	LC–MS/MS and Western blot validation	Fifty proteins were differentially abundant between the two groups. Proteins involved in cytoskeleton and immunity were more abundant in the immune castrated group. Several heat shock proteins (HSPs) and laminins were abundant in the surgical castrated group.	[38]
	Surgical castration against entire male pigs	LD	2-DE and MALDI-TOF/TOF	Entire male pigs have a more oxidative metabolic profile than surgically castrated pigs. More abundance of structural protein fragments suggests a higher degree of proteolysis in entire male pigs.	[39]
Growth	Muscle growth and lipid deposition. Comparison between two Chinese mini-type breeds and two western breeds.	LD	iTRAQ, LC-MS/MS	288 differentially abundant proteins of which 169 were upregulated and 119 were downregulated between the two types of genotypes. Among them, 28 were related to muscle growth and 15 to lipid deposition.	[51]
Animal welfare	Animal stress and welfare changes in the housing system	Blood	2D-DIGE and MALDI-TOF/TOF iTRAQ	Changes in two main homeostatic mechanisms: the innate immune and redox systems. The acute phase proteins haptoglobin, apolipoprotein A-I and α1-antichymotrypsin 3 (SERPINA3), and the antioxidant enzyme peroxiredoxin 2 were differentially expressed.	[50]
	Heat stress. Effect of chronic heat stress, thermal neutral and restricted feed intake conditions on hepatic proteomes	Liver	2-DE and LC-MS/MS	Forty-five hepatic proteins were differentially abundant among groups. The proteins were involved in response to stress and immune defense, oxidative stress response, cellular apoptosis, energy metabolism, signal transduction, and cytoskeleton.	[43]
	Heat stress. Effect of chronic heat stress against thermal neutral on meat quality	LD	2-DE and LC-MS/MS	Changes in the expression of myofibrillar proteins, glucose and energy metabolism-related proteins, heat shock proteins, and antioxidant enzymes might be affecting tenderness.	[44]
	Animal welfare (heat stress) Effect of acute heat stress for 2, 4 and 6 h against thermal neutral on meat quality	ST (red and white areas)	2-DE and Western Blot	Several proteins were altered affecting metabolism, cell structure, chaperone, antioxidant, and proteolytic activity. The proteome data showed that the muscle proteome was altered from 2 h of heat stress.	[46]
	Animal stress (lysine acetylation)	LD	Acetylpeptide enrichment and LC-MS/MS	The acetylation of proteins was enriched in muscle contraction, carbohydrate metabolism, cell apoptosis and calcium signaling.	[49]

LD = Longissimus dorsi, ST = Semitendinosus.

## 3. Application of Proteomics to Assess Pork Quality and Authentication

### 3.1. Influence of Different Post-Mortem Traits on Pork Quality

Meat quality is generally determined by farm production factors as well as by post-mortem interventions techniques applied at the industrial scale. Recent studies regarding the effect of different post-mortem traits aiming to assess and improve pork quality are shown in Table 2. Within pork quality, tenderness is the main important sensory quality trait that is mainly affected by connective tissue, intramuscular fat content, and myofibrillar structure, which may interact with storage time [52]. In the early stages of rigor mortis, meat tenderization is widely studied from a proteomic point of view. In this process, protein degradation due to the muscle enzymes, pH decline, and tissue oxidation are key factors in developing tenderness, flavor, color, and juiciness. In the case of protein oxidation, relevant biomarkers for the oxidative process are the group of peroxiredoxins that control the hydrogen peroxide concentration to protect cells. Indeed, peroxiredoxin-2 (PRDX2) was found to be more abundant in tenderer pork loin during the aging [53]. Furthermore, PRDX3 and PRDX6 were found to be significantly correlated with the lightness (L*) and redness (a*) color traits, respectively [54,55]. Other oxidative stress proteins were found to be related with pork color such as Parkinson disease protein 7 (PARK7), playing a pivotal role in cell protection against oxidative stress and cell death [55]. During the processing and storage of products, protein oxidation contributes to reduce pork quality [56]. For this reason, the storage technologies of pork were assessed to avoid protein oxidation. For instance, in the case of high-oxygen atmospheres, the disulfide bonds among myosins are formed as a result of cysteine and methionine oxidation. Additionally, the oxidation of both amino acids produces different oxidation products at different sites, representing a high complexity [57].

In the case of pork quality, proteomic technologies have been demonstrated to be very effective in the prediction of drip loss and ultimate pH from 50% to 80% of protein biomarkers in the total proteome [58]. This trend of prediction was further confirmed by Kwasiborski and co-workers on these quality traits as well others such as color parameters [55]. This means that proteomics is a promising approach to efficiently assess pork quality. An intense change of proteome was observed and associated with pH, color, and drip loss traits, resulting in 140 differentially expressed proteins. Functional analysis showed a decreased release of Ca^2+^, lower contents of type II fibers and those of glycogen, which decreased the extent of glycogenolysis in high-quality meats from the *longissimus dorsi* muscle [21]. Considering only drip loss, an enrichment analysis resulted in sphingolipid metabolism and glycolysis/gluconeogenesis as key pathways significantly influencing drip loss [59]. However, water holding capacity trait over post-mortem aging is not yet fully understood by the preliminary proteomic studies and further work is needed [60]. On the other hand, intramuscular fat content positively influences the taste and is both regulated by adipogenesis and myogenesis, which balances the number and the size of adipocytes and myocytes [61]. A strategy to reduce the intramuscular fat is by lowering the use protein diets, which seems to be at the origin of the enhancement of glycolysis and the Krebs cycle pathways as well as modifications in mitochondria, contractile proteins, and calcium signaling, therefore, impacting the extent of the glycolytic to oxidative properties of fibers [62]. It is well-known that in pork quality, the fiber distribution of oxidative and glycolytic types (red and white muscles) is a key factor in post-mortem metabolism [63]. Myosin-1, myosin-4, troponin complex (fast), myosin light chains, and metabolic enzymes are overexpressed in glycolytic fibers and myosin-2, myosin-7, and myoglobin; meanwhile, mitochondrial oxidative metabolic enzymes were especially abundant in oxidative fibers [64]. The high amount of oxidative fibers suggests, at first glance, an increased quality due to the link with a higher amount of intramuscular fat.

### 3.2. Proteomics and Authentication/Adulteration of Pork Products

In addition to the above-mentioned, other objective of the pork industry is food authentication and the detection of adulterations. A non-extensive summary of pork product authentication/adulteration is shown in Table 2. The replacement of ingredients with undeclared species, animal tissues, and geographic origin or the distinction of non-ecological meat and freeze/thawed meat is a technical challenge for proteomics. This is a complex issue because proteins undergo chemical modifications such as Maillard reactions in highly processed food products, hampering efficient authentication. In animal species identification, a set of heat-stable peptide markers from myosin, myoglobin, hemoglobin, L-lactase dehydrogenase A, and β-enolase allows for the preliminary authentication of eleven types of white and red meats from chicken, duck, goose, turkey, pork, beef, lamb, rabbit, buffalo, deer, and horse [65]. Despite the fact that immunoassay techniques (ELISA) are often used to quantify target compounds, some disadvantages are likely to be the low efficiency and cross-reactivity between species, making more difficulties and hindering identifications. For example, the skeletal muscle protein troponin I detection was quantifiable from 8.7 to 52 ng/mL by the ELISA method, and similar results were achieved by 2-DE in combination with Matrix-Assisted Laser Desorption/Ionization-Time-Of-Flight (MALDI-TOF/TOF) [66]. In this regard, mass spectrometry becomes a relevant method to quantify specific peptides of different species that could be proposed as biomarkers. For example, five peptides from myosin were used to identify and quantify trace pork (up to 0.5%) in meat mixtures by parallel reaction monitoring (PRM) [67]. On the other hand, pork products and unprocessed meat should be packed, distributed, and stored appropriately. Fresh meat is more appreciated than frozen meat because of the formation of ice crystals than can destroy the ultrastructure of the matrix and consequently denature the proteins, hence reducing the potential meat quality. By using meat exudates to search for protein biomarkers, it was possible to discriminate between fresh and frozen meat [68]. From this study, a total of 22 proteins were found as candidate discriminatory biomarkers using gel-based techniques.

**Table 2:** Recent studies regarding the effect of different post-mortem traits aiming to assess and improve pork quality as well as studies related to food safety and pork product authentication/adulteration.

Studied Factors		Protein Source	Proteomic Approach	Main Findings	Ref
Ageing	Ageing pork. Effect of protein lysine acetylation in post-mortem muscle changes	LD	Acetilpeptide enrichment and LC-MS/MS	Acetylproteins involved in apoptosis, calcium signaling, and IMP synthesis were identified in post-mortem porcine muscle. The lysine acetylation of proteins regulate the conversion of muscle into meat.	[47]
	Tenderness and aging	LD	2D-DIGE and mass spectrometry	Soluble desmin and peroxiredoxin-2 could be used as biomarkers of tenderness in aged pork products.	[53]
	Protein oxidation (oxidation of cysteine and methionine residues) Effect of hydroxyl radicals on the myosin	LD	SDS-PAGE, cysteine and methionine labelling and LC-MS/MS	The cysteine at the head of the myosin and that at the coiled tail of myosin easily generated disulfide. Further, the methionine at the coiled tail of myosin was more easily oxidized than that of the head.	[57]
Pork quality	Effect of proteome profiles on meat quality (high-quality samples against low-quality)	LD	Tandem mass tag labelling and mass spectrometry	Lower degree of glycolysis in high-quality compared to low-quality meat. The levels of oxidative stress and apoptosis were low in high-quality meat.	[21]
	Meat quality: drip loss Identification of candidate genes	LD	Isotope coded protein labelling followed by selected reaction monitoring analysis	The enrichment analysis resulted in 10 pathways. The most relevant pathways were sphingolipid metabolism and glycolysis/gluconeogenesis in relation to drip loss. It allowed proposing genetic markers and candidate genes for drip loss.	[59]
	Meat quality: pH, color traits, drip loss, water holding capacity	LD	2DE + MALDI-TOF	Proteins associated with ultimate pH, lightness, drip, thawing and cooking loss were related to the glycolytic pathway, phosphate transfer, or fiber type composition. In the case of thawing loss, the proteins were related to denaturation of myofibrils or lipid content. Redness involved proteins were enriched in post-mortem oxidative activity.	[55]
	Intramuscular variation, neat quality (color, drip loss and tenderness) and their relation to proteome	LD	Label-free quantification + LC-MS/MS	Glycolysis enzymes (enolase 3, ALDOA, LDHA, PGM1, and TPI1) were highly abundant in the medial and posterior region. GAPDH and myoglobin were overexpressed in the medial region	[69]
	Water holding capacity measured as centrifugal exudate (High drip vs. Low drip) across post-mortem aging on different phenotypes	LD	2-D DIGE followed by MALDI-TOF/TOF and nano-ESI LC-MS/MS.	Discriminatory proteins identified include metabolic enzymes, stress response, transport and structural proteins. Twenty-five proteins were used to discriminate between high drips and lower drips with accuracy higher than 72%.	[60]
	Intramuscular fat content	LD	Tandem mass tag labelling and parallel reaction monitoring analysis	ALDH1B1, OTX2, ANXA6 and Zfp512 were proposed as candidate biomarkers associated with intramuscular fat deposition and fat biosynthesis in Laiwu pigs.	[70]
	Muscle fiber type distribution in semimembranosus and semitendinosus muscles	SM, ST separated into dark and light portion	LC-MS/MS	According to fiber type (oxidative vs. glycolytic) distribution, differentially expressed muscle proteins was detected resulting in intramuscular variations of pork quality.	[64]
	Effect of feeding regime on intramuscular fat increase. Comparison between normal protein diet vs. reduced protein diet.	LD	iTRAQ and LC-MS/MS	The categories “muscle contraction” and “structural constituents of cytoskeleton” were the most significantly up-regulated proteins in muscle from reduced protein diets and up-regulated proteins involved in the regulation of energy metabolism.	[62]
Mislabeling	Authentication of pork in meat mixtures (chicken, sheep and beef)	Meat mixtures	Parallel reaction monitoring mass spectrometry	Five peptides from myosin were screened and then used for pork detection by PRM of Orbitrap MS. The LOD in mixed meat can be up to 0.5%.	[67]
	Adulteration. Search for species-specific biomarker of mammalian muscle tissues in raw meat and meat products.	Meat mixtures	2-DE and MALDI-TOF/TOF	Troponin I (TnI) has been characterized as a potential thermally stable and species-specific biomarker of mammalian muscle tissues in raw meat (beef, pork, lamb, and horse) and meat products.	[66]
	To discriminate fresh and freeze-thawed pork	LD	2-DE and MALDI-TOF/TOF	Twenty-two proteins were discrimination markers for fresh or and freeze-thawed pork.	[68]
Food safety	Prevention and control of *Salmonella typhimurium* in pigs along a time course of 1, 2, and 6 days post infection	Intestinal sections (ileum and colon)	iTRAQ	The expression changes in colon were found in proteins involved in cell death and survival, tissue morphology or molecular transport at the early stages and tissue regeneration at 6 days post-infection. A higher number of changes in protein expression was quantified in ileum at 2 days post-infection	[71]

SM = semimembranosus muscle ST = semitendinosus muscle.

## 4. Advances in Proteomics for Pork Products

Nowadays, pork is an important part of the diet of many cultures because of its great versatility and thee abundant foods that can be manufactured from sausage to a dry-cured ham. Indeed, in recent years, most pork meat is sold as ham, bacon, and sausages than fresh pork. Further processing of pork should be considered in great detail to achieve high quality and palatability of products. The quality is associated, as evidenced above, with protein structure and lipid and protein oxidative reactions occurring during industrial processing and storage. Therefore, proteomics emerges as a relevant field, giving rise to new knowledge and understanding of the mechanisms. Table 3 displays the recent studies regarding the use of proteomics/peptidomics to evaluate the quality of pork products.

### 4.1. Technological Properties of Proteins

Regarding the functional properties of proteins, sausages contain a major insoluble protein fraction with a high content of myofibrillar proteins compared to cooked hams. This insoluble protein fraction is rich in tropomyosin and myosin light chains forming aggregates with actin. Moreover, a high level of protein oxidation was identified due to oxidized methionine in cooked products. The myofibrillar network, together with coagulated sarcoplasmic protein matrices, establishes the gel properties of cooked pork products [72,73]. Salt is an essential ingredient for the elaboration of pork products, providing interesting flavors and tastes, and increasing their shelf life [74]. For this reason, a large part of pork products is marketed as cured products such as bacon and dry cured-ham. In the case of the latter product, low water activity (<0.90) and the use of sodium chloride and nitrite allows for the production of a cured meat product with better stability. These conditions of the food matrix determine the protein hydrolysis, and consequently, the flavor and textural properties are very dependent on the intensity of proteolysis during the dry-cured ham ripening [75]. The major fraction of muscle proteins is the myofibril, responsible for muscle structure. It was demonstrated that proteolysis indices and adhesiveness were correlated to the degradation of myofibrillar proteins. Additionally, it was reported that α-actin, myosin-1, and myosin-4 could be used as protein biomarkers of proteolysis and adhesiveness [76]. 

Technical improvements were analyzed from a proteomic point of view, providing an insight into the molecular modifications occurring in the meat matrix. Regarding healthy pork products, reducing salt content without changing the sensory and textural properties is the main goal in the production of dry-cured ham. For instance, when a pressure treatment before salting was investigated, glycolytic enzymes as well as myofibrillar proteins were found differentially abundant in exudates, suggesting a faster loosening of the myofibrillar structure [77]. Other strategies to reduce salt content assayed in dry-cured ham are ultrasounds or high-pressure processing. High-pressure conditions caused proteolysis in dry-cured ham, releasing two important amino acids (leucine and isoleucine) in terms of their role in sensory traits in a significant concentration. This fact could be assessed measuring the secondary metabolites 2-methyl-butanal and 3-methyl-butanal, which are related to the proteolysis process [78]. Other emerging technologies were the use of ultrasounds on the sliced dry-cured ham to correct texture defects. We found that ultrasound thermal treatment strongly increased the degradation of proteins, specifically those of the myofibril [79]. Additionally, sarcoplasmic proteins showed an increased abundance due to ultrasound treatment, suggesting that they could be further used as biomarkers to control the impact of ultrasound process. 

### 4.2. Bioactive Peptides from Pork Products

Beyond this technological aspect, the bioavailability and bioaccessibility of food proteins are key factors of meat products to assess their nutritional and healthiness potential [80]. These food peptides from the entire protein can reach the intestine as peptides or be liberated after digestion. These biopeptides have been demonstrated to have antidiabetic, cholesterol-lowering, antihypertensive, anticancer, antimicrobial, or antioxidant activity in different in vitro experiments [81,82]. However, it should also be considered that for the development of these new functional products, these small peptides and free amino acids influence the flavor and aroma of final products due to their bitterness [78]. In addition, their low bioavailability is the main problem that limits their utilization in functional foods. This fact is often associated with their selective intestinal uptake and physiological instability when peptides are taken orally, hence it is crucial to select adequate processing methods and less reactive matrices when producing biopeptides to maintain their structures and improve their bioaccessibility and bioavailability [82]. The amino acids and small peptides (di and tri-peptides) can be absorbed more easily during digestion, however, the food processing may produce Maillard compounds, oxidation of sulfur amino acids, and other side reactions that reduce the protein digestibility and quality. Additionally, the protein denaturation and subsequent aggregation contribute to a low bioavailability [83]. In this sense, peptidomics could be used as a tool for assessing the bioactivity of peptides and their relation to human health [84]. 

In the case of dry-cured ham, titin is an important protein responsible for muscle structure and is involved in the connection between actin and myosin. The degradation of this protein was assessed over the curing process, and titin-derived peptides (KDEAAKPKGPIKGVAKK, KKLRPGSGGEK, KNTDKWSECAR and ISIDEGKVL) have been proposed as candidate markers for processing time [85]. Moreover, the degradation of structural proteins (myosin, α-actinin, and troponin-T) and creatine kinase muscle type have been associated with the bitterness and adhesiveness of Jinhua ham. More in-depth, it has been demonstrated that cathepsin B and B + L are primarily responsible for the bitterness and adhesiveness, together with excessive proteolysis [86]. As indicated above, protein oxidation is mainly behind the quality deterioration of cured products, as was demonstrated using a peptidomic approach. Effectively, most peptides of dry-cured hams were oxidized on the methionine site in their sequence. In this sense, peptides from the major myofibrillar proteins (nebulin, titin, myosin heavy chain, and troponin I) were the main oxidized entities [87].

The hydrolysis of food proteins and modifications are produced as a result of the cooking process. In the case of pork, a high cooking temperature leads to a reduction in digestibility with significantly different peptidomes. It seems that sites of trypsin cleavages remain more stable with temperature than pepsin cleavages [88]. This demonstrates that the cooking process has a great impact on protein digestibility. Moreover, the degree of salt in pork soup affected the protein modifications as confirmed by the fact that the proteolytic index was higher (5%) in soup compared to the unsalted product (2%). iTRAQ technology, which utilizes isobaric reagents to label the primary amines of peptides and proteins, revealed that 13 collagen proteins were differentially abundant in soup (2% salt) in comparison to soup without salt [89]. This means that the cooking times and temperature as well as other ingredients provoke changes in protein and peptide fraction and digestibility. 

On the other hand, the search for bioactive peptides from vegetable and animal products is also carried out in cured pork products [90]. Bioactive peptides produced by gastrointestinal digestion or enzymatic hydrolysis and during food processing were evidenced [91]. In the case of curing, it was proved that peptides (<1 kDa) from dry-cured Xuanwei showed more antioxidant activity than Jinhua ham. A high scavenging effect against O^2−^ was measured by the 2,2-diphenyl-1-picrylhydrazyl (DPPH) method by the hydrolyzation of myosin, troponin, and actin [92].

**Table 3:** Recent studies regarding the use of proteomics/peptidomics to evaluate the quality of pork products.

Product	Objective	Proteomic Technology	Main Findings	Ref
Cooked pork products (cooked ham and emulsion sausages)	Effect of cooking process on protein modifications	2-DE and MALDI-TOF/TOF	The protein aggregation systems of cooked hams and emulsion sausages reflect the heat processing conditions. The disulfide bridges and additional covalent interprotein links determine the final product.	[72,73]
Parma dry-cured ham	Effect of pressure treatment before salting stage	2D-PAGE and LC−ESI-MS/MS	Specific proteins were found differentially abundant in exudates from pressed versus unpressed hams. The pressure caused a faster loosening of the myofibrillar structure with the release of specific groups of proteins	[77]
Dry-Cured Ham	Effect of Proteolysis indices and adhesiveness on proteins degradation Use of high pressure and ultrasound to correct textural defect in dry-cured ham	2-DE and MALDI-TOF/TOF	Myosin-1, α-actin and myosin-4 proteins were the main changing due to proteolysis. The high-pressure conditions caused a greater level of proteolysis displaying that actin was differentially degraded, unlike myosin. Fragments of the major myofibrillar protein were abundantly caused by ultrasound heating.	[76,78,79]
Dry-cured ham (Jinhua)	Sensory attributes (formation mechanisms of bitterness and adhesiveness) in raw, normal and defective hams	LC-MS/MS	Defective hams showed more proteolytic index that normal ham. Creatine kinase, myosin, α-actinin and troponin-T showed the most intense response to bitterness and adhesiveness of dry-cured ham. Myosin was proposed as a suitable biomarker to monitor bitterness and adhesiveness	[86]
Dry-cured ham	Peptide oxidation in PDO Teruel dry-cured ham	nESI-LC–MS/MS	KDEAAKPKGPIKGVAKK, KKLRPGSGGEK, KNTDKWSECAR and ISIDEGKVL were proposed as peptide biomarkers of processing conditions.	[87]
Cooked pork	Effect of cooking on peptidomic profile and digestibility	SDS-PAGE and MALDI-TOF/TOF	The cooking process led to a reduction in digestibility. Peptides sequenced from pepsin-digested samples under lower degrees of doneness disappeared as the temperature increased. The trypsin cleavages appeared more consistent among different degrees of cooking	[88]
Pork soup	Protein modifications in presence of salt (treated 2%) and without salt (control)	i-TRAQ	Proteolytic index of salted samples was 5% higher than the control and 112 differentially abundant proteins were detected.	[89]
Dry-cured ham	Antioxidant peptides from Xuanwei (XHP) and Jinhua (JHP) ham	nano-LC-MS/MS and quadrupole ion trap Orbitrap spectrometer	XHP showed higher antioxidant ability than JHP. The oligopeptides with less than 1000 Da and high antioxidant activity were detected.	[92]
Dry-Cured Ham	Degradation of sarcoplasmic proteins	nLC–MS/MS and SDS-PAGE	Twenty proteins were identified and quantified suggesting intense degradation during processing.	[93]

## 5. Conclusions and Future Prospects 

Proteomics is an emerging technology for the rapid and sensitive identification of biomarkers aiming to assess the potential quality of pork products and the impact of food processing technologies. Genetic and rearing conditions influencing technological and sensory meat quality provoke different biochemical and molecular reactions that are regulated by several proteins and pathways including metabolic enzymes. Furthermore, pork quality determined by tenderness, color, drip loss, and intramuscular fat is conducted by structural and sarcoplasmic proteins. In this regard, proteins play a key role in the textural and sensory quality of pork fresh, showing the importance of the study of muscle proteome in pork. The most relevant quality traits were assessed by gel and mass spectrometry analysis. Gel-based proteomics are widely used for the search of protein biomarkers of these quality traits. Even the most sensitive gel-based methods such as protein labeling with fluorescent dyes as fluorescence difference gel electrophoresis (DIGE) were considered. However, gel-free alternatives such as Sequential Window Acquisition of All Theoretical Mass Spectra (SWATH-MS), Liquid chromatography–mass spectrometry/mass spectrometry (LC-MS/MS), or Isobaric tags for relative and absolute quantitation (iTRAQ) should be employed to enhance the efficiency of our quest for protein biomarkers and further validate previous results. Other technical improvements in pork processing were assessed from a proteomic perspective, providing an insight into protein modifications. Peptidomic profiles could further offer an overall overview of the protein digestibility and bioavailability that determine the effect of protein fraction on human health. In the framework of data analysis, we expect that statistics will play a great role in future proteomics, especially in handling the huge data produced by different proteomics methods. Regarding this, we expect that there is great interest in combining different omics techniques in the framework of multi-omics to study the interplay between different macromolecules in relation to the pork phenome. Indeed, phenomics or high-throughput phenotyping is becoming a reality in livestock production systems including pork, and we expect that this global approach will play an important role in the next years and decades.
